# Supplementary material for: Pyruvate Dehydrogenase Inhibition Leads to Decreased Glycolysis, Increased Reliance on Gluconeogenesis and Alternative Sources of Acetyl-CoA in Acute Myeloid Leukemia
Source: Cancers (Basel). 2023 Jan 12;15(2):484. doi: 10.3390/cancers15020484 (PMC9857304; doi:10.3390/cancers15020484)
Supplement: Supplementary file 1 [file cancers-15-00484-s001.zip › cancers-2075375-supplementary.pdf]

Supplemental Material

Figure S1

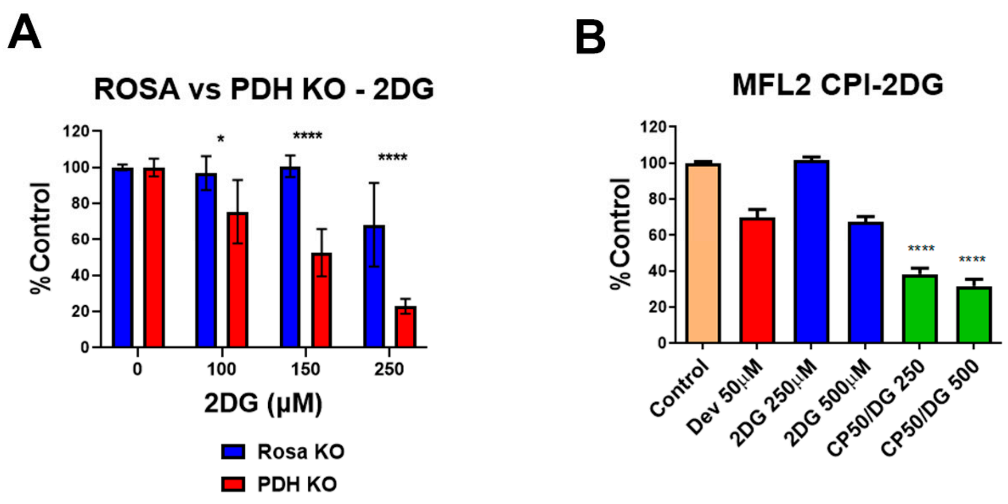

Figure S1: TCA cycle inhibition engenders reliance on glycolysis. A) ROSA control or PDH deleted cells were treated with the indicated amount of 2-deoxy-D-glucose (2DG) for 72-hours and viability was assessed. B) MFL2 cells were exposed to the indicated doses of devimistat (Dev), 2DG or the combination for 72-hours and assessed for viability. **** = $p < 0.0001$, * = $p < 0.05$
