# Supplementary figures and images for: Pyruvate Dehydrogenase Inhibition Leads to Decreased Glycolysis, Increased Reliance on Gluconeogenesis and Alternative Sources of Acetyl-CoA in Acute Myeloid Leukemia
Source: Cancers (Basel). 2023 Jan 12;15(2):484. doi: 10.3390/cancers15020484 (PMC9857304; doi:10.3390/cancers15020484)

**Figure 1F**

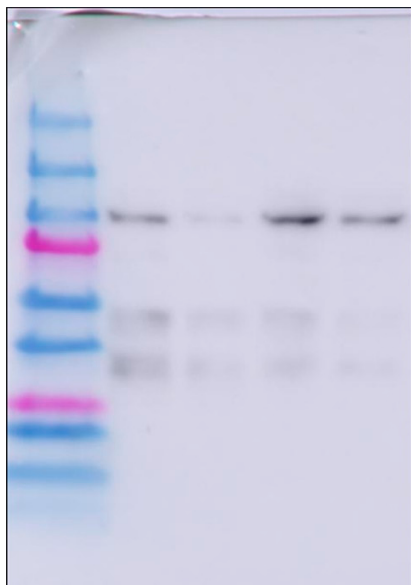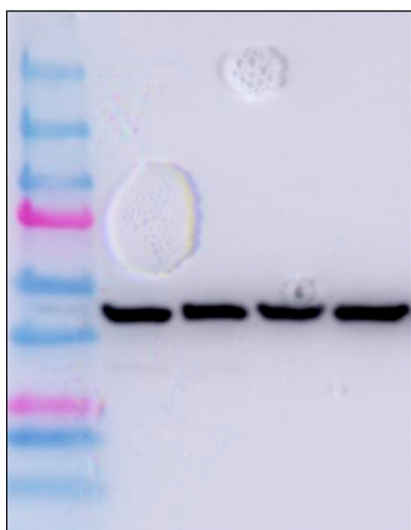

**Figure 1G**

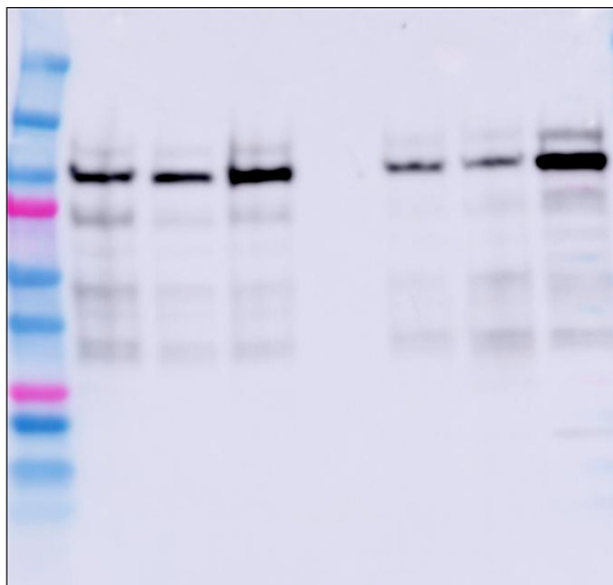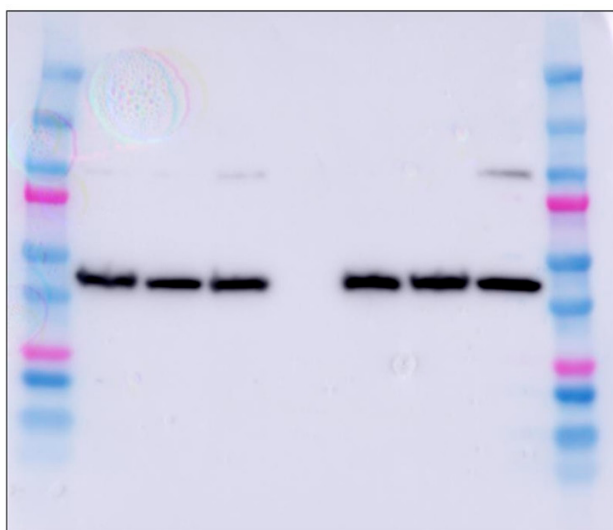

**Figure 2**

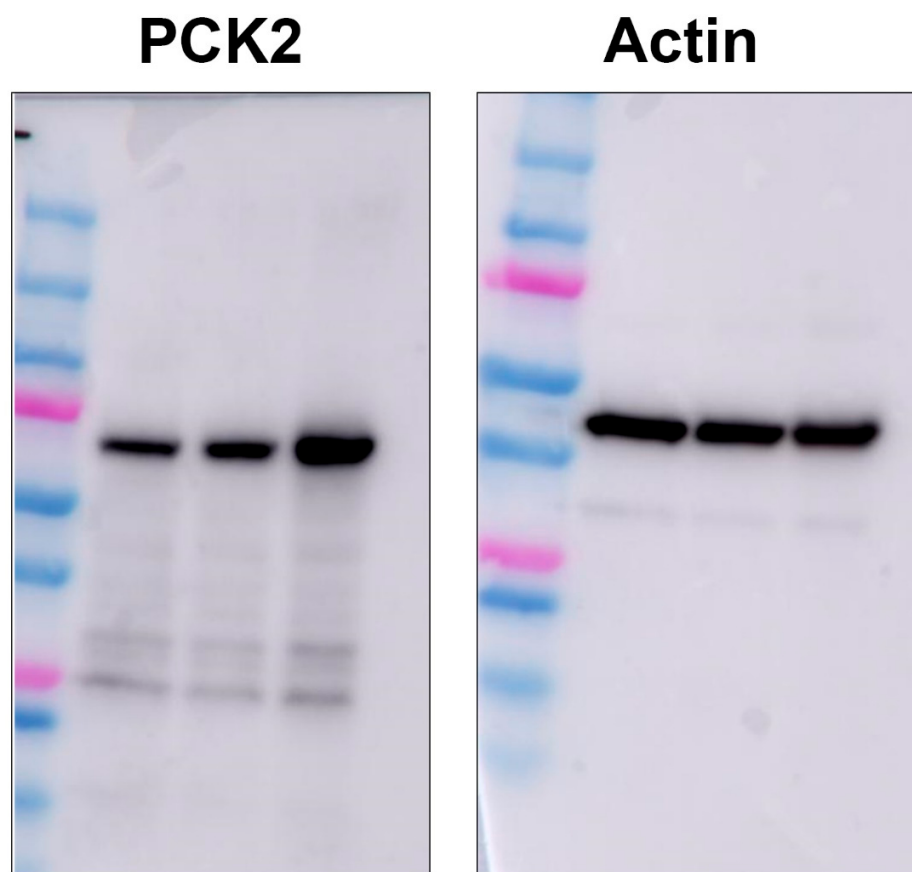

**Figure 4C**

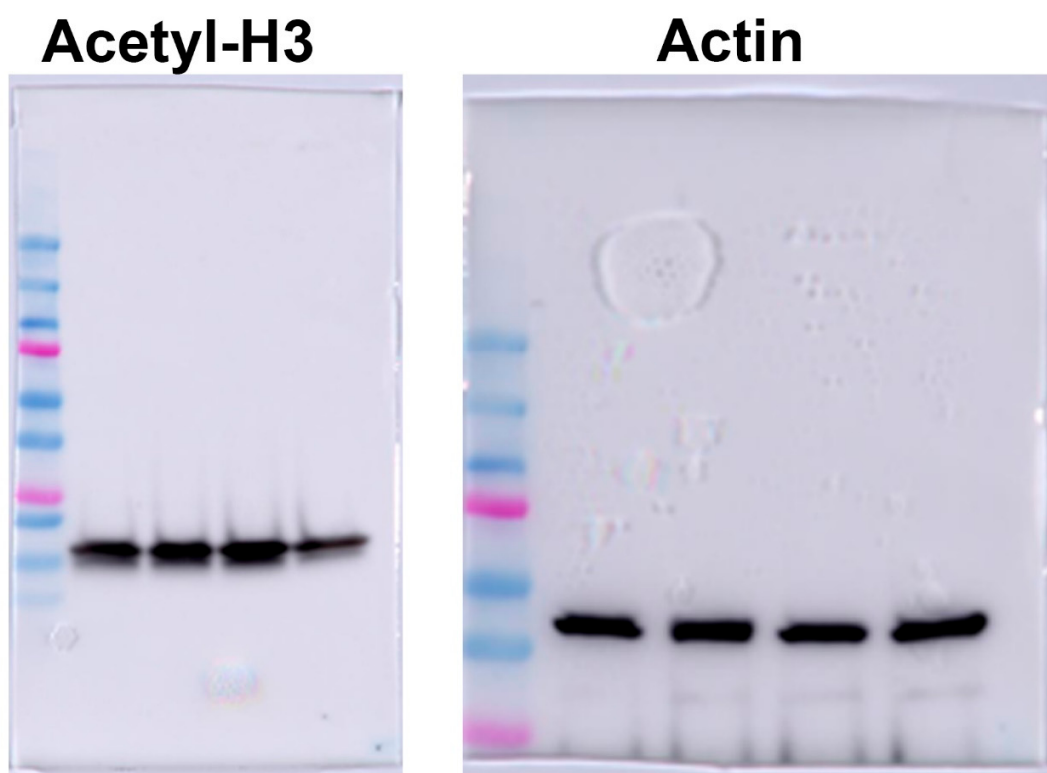

Supplement: Supplementary file 1 [file cancers-15-00484-s001.zip › File S1.pdf]
